# Predominance of reassortant infectious bursal disease viruses in Turkish poultry flocks

**DOI:** 10.1016/j.psj.2025.105974

**Published:** 2025-10-20

**Authors:** Erhan Bayraktar, Ozge Aydin, Hasan Emre Tali, Ismail Egemen Ozkan, Sajid Umar, Hamid Besim Tali, Aysun Yilmaz, Nuri Turan, Ahmet Kolukisa, Cem Konuk, Altug Erdem, Abubakar Siddique, Munir Iqbal, Juergen A. Richt, Huseyin Yilmaz

**Affiliations:** a Poultry Division, CEVA Animal Health, Maslak, Turkey; b Department of Virology, Veterinary Faculty, Istanbul University-Cerrahpasa, Buyukcekmece, Hadimkoy, Istanbul, Turkey; c Global Health Research Center (GHRC), Duke Kunshan University, China; d Division of Natural & Applied Sciences (DNAS), Duke Kunshan University, China; e College of animal Sciences, Department of veterinary medicine, Zhejiang University, Hangzhou, China; f The Pirbright Institute, Ash Road, Pirbright, Woking, GU24 0NF, UK; g Department of Diagnostic Medicine and Pathobiology, College of Veterinary Medicine, Kansas State University, Manhattan, USA

**Keywords:** Infectious bursal disease virus, Genetic diversity, Phylogenetic analysis, Reassortment, Türkiye

## Abstract

Infectious bursal disease (IBD), caused by the infectious bursal disease virus (IBDV), is an acute, highly contagious viral infection that severely immunosuppresses poultry, threatening global poultry health and production. The co-circulation of field and vaccine strains of IBDV in poultry flocks facilitates genetic diversification through mutation and recombination, leading to the emergence of novel variants. To assess this dynamic, we characterized the genetic diversity of IBDV strains in vaccinated Turkish flocks during 2024–2025. RT-PCR detected IBDV in 91.4 % of broiler and 90.2 % of layer samples. Genetic analysis of the VP2 and VP1 genes from 35 strains identified three co-circulating genotypes. Notably, reassortant A3B1 strains were predominant, while classical (A1B1) and very virulent (A3B2) strains were less common, highlighting reassortment as a key driver of current IBDV evolution. The majority (*n* = 30) clustered within the A3 genogroup, sharing 89.38–99.57 % VP2 identity with reference strains. Critically, amino acid analysis confirmed that all A3 strains carried the key amino acid markers of very virulent IBDVs (222A, 242I, 253Q, 256I, 279D, 284A, 294I, 299S). In contrast, the single A1 strain exhibited a classical signature (222P, 249Q, 286T).

This study represent a comprehensive report on the emergence and dominance of reassortant A3B1 strains in Turkish poultry, providing critical insights into the genomic and antigenic evolution of IBDV. These findings indicate that novel preventive and vaccination strategies for emerging reassortant IBDVs are needed.

## INTRODUCTION

Numerous viral infections like infectious bursal disease, Newcastle disease, avian influenza and infectious bronchitis have a devastating effect on poultry healt. Infectious bursal disease (IBD) is a highly contagious viral disease of chickens, leading to acute mortality, immunosuppression and nephrosis (Müller et al., 2012;Alkie and Rautenschlein, 2016;Islam et al., 2021). The disease primarily targets the bursa of Fabricius, a key lymphoid organ, causing severe lesions and destruction of immature B-lymphocytes. Chickens aged 3–6 weeks when the bursa is most developed are particularly susceptible. Besides, older chickens are also susceptible to IBDV infections with a lesser frequency (Orakpoghenor et al., 2020a). Clinical signs include depression, diarrhea, dehydration, and sudden deaths. Post-mortem findings often show swollen, hemorrhagic, or atrophied bursae. Vaccination failure can result from prolonged immunosuppression, which creates favorable conditions for secondary infections by opportunistic avian pathogens (Jackwood et al., 2018;Mosad et al., 2024). The disease poses significant economic losses due to high mortality, reduced vaccine efficacy, secondary infections and impaired growth. Control of IBDV relies on strict biosecurity, vaccination and proper flock management.

The etiological agent of IBD, infectious bursal disease virus (IBDV), is a non-enveloped, icosahedral virus approximately 60 nm in diameter. It possesses a double-stranded, bi-segmented RNA genome (segment A and segment B) and is classified within the genus *Avibirnavirus* of the family *Birnaviridae* (Delmas et al., 2019)*.* Segment A (3.2 kbp) contains two open reading frames (ORFs). The small ORF encodes the non-structural viral protein VP5, which facilitates viral egress. The large ORF produces a polyprotein that is post-translationally processed to generate three structural proteins: VP2 (the major capsid protein), VP4 (a protease), and VP3 (a multifunctional protein that binds viral dsRNA and mediates genome-capsid interactions). Segment B contains a single ORF encoding the RNA-dependent RNA polymerase (VP1), responsible for viral replication (Alkie and Rautenschlein, 2016;Méndez et al., 2017;Reddy et al., 2024).

IBDV was first isolated in the United States in 1957 and was initially characterized as the classic strain (Müller et al., 2003). IBDV comprises two distinct serotypes: Serotype I, which is pathogenic to chickens and causes clinical disease, and Serotype II, which is avirulent in chickens (Müller et al., 2003). IBDV field strains are traditionally classified into 5 major pathotypes: 1: Classical virulent strains, 2: Antigenic variant (v) strains, 3: Novel variant (nv) strains, 4: Very virulent (vv) strains and 5: Attenuated strains. The pathogenicity of these strains is influenced by both genomic segments A and B (Müller et al., 2003). Since its discovery, the virus has spread globally and undergone significant evolutionary diversification. The subsequent emergence of antigenic variant strains and very virulent IBDV (vvIBDV) strains has presented new challenges for both outbreak control and vaccine development. Since 1957, classical virulent strains remain in circulation and typically induce immunosuppression accompanied by clinical signs. The antigenic variant strains that emerged in 1980s U.S. outbreaks often cause subclinical infections but still lead to significant immunosuppression, compromising vaccine efficacy and predisposing birds to secondary infections. In contrast, vvIBDV strains (also emerging in the 1980s) provoke acute disease with high mortality rates. Notably, live vaccine strains may also be detected in commercial flocks alongside these field variants(Huang et al., 2022; Reddy et al., 2024). The new variants and reassortant IBDVs have been reported in recent years (Pikuła et al., 2018;Pikuła et al., 2020;Gao et al., 2023).

The VP2 capsid protein represents the principal immunodominant antigen of IBDV and serves as the primary target for neutralizing antibodies. A critical hypervariable region (HVR, aa 220-330) within VP2 undergoes intense immune selection pressure, resulting in significant antigenic drift. This region contains four key hydrophilic loops (P-BC, P-DE, P-FG, and P-HI) that govern viral virulence and determine neutralization profiles (Alkie and Rautenschlein, 2016). Due to its variability, the VP2-HVR has become the standard marker for molecular characterization, with extensive sequence data available in public databases (Islam et al., 2021).Continuing antigenic evolution has led to the classification of global IBDV strains into distinct genogroups based on HVR sequence diversity. Current systems recognize up to nine genogroups with some variation among classification schemes. Wang et al. (2021) differs primarily in classifying attenuated strains as A8 while Gao et al. (2023) groups attenuated strains as A9. While consensus exists regarding the major genogroups, ongoing refinements to the classification system reflect the continuous evolution and geographic diversification of IBDV strains (Wang et al., 2021; Islam et al 2021; Gao et al., 2023; Reddy et al., 2024).

Epidemiological studies often target the VP1 gene to further classify IBDV. VP1, encoded by segment B, serves as a basis for categorizing IBDV strains into five distinct genogroups (B1 to B5) (Islam et al., 2021). Current typing systems classify strains based on both A and B genogroups for instance, classical strains are designated as A1B1, US variants as A2B1, and very virulent (vv) strains as A3B2. The segmented nature of the IBDV genome allows reassortment to occur in co-infected birds, resulting in progeny viruses with mixed A and B segments from different parental strains. Consequently, molecular epidemiology studies must sequence both genome segments to detect such events. Reassortant strains have been reported globally, involving exchanges between different genogroups and even serotypes. Notably, in 2020 and 2023, reassortant IBDV strains carrying a vv segment A and a non-vv segment B (genogroup A3B1) were found to be widespread in European chicken flocks. Some of these strains also exhibited antigenic drift, accumulating HVR mutations and forming a distinct Western European clade (Gao et al., 2023; Pikula et al 2018; Pikula et al., 2020; Reddy et al., 2024).

In Türkiye, IBD was first identified in a broiler flock in 1978 and has since caused significant economic losses to the poultry industry (Coven, 1999a;Ture, 1999b; Sareyyüpoğlu and AkanRestriction, 2006;Yilmaz et al., 2019;Kurtbeyoğlu and Akan, 2024). Although IBD is a significant threat to poultry health in Türkiye, data on the genetic diversity of IBDV strains circulating in the country remain limited. Recent studies by Yilmaz et al. (2019) and Kurtbeyoglu and Akan (2024) have confirmed the circulation of classical, vvIBDV, and reassortant IBDV strains in Turkish poultry flocks (Yilmaz et al., 2029; Kurtbeyoğlu and Akan, 2024). Despite the widespread use of IBDV vaccines globally, the emergence of novel variants and reassortant IBDVs continues to pose a threat to poultry health and production, leading to substantial economic impacts worldwide.

Molecular techniques have revolutionized poultry disease management by enabling highly accurate, rapid, and sensitive pathogen detection. Unlike conventional methods (e.g., culture, serology), which can be time-consuming and less specific, molecular tools help minimize false results, reduce financial losses, and enhance disease management (Ardicli O; Demirbilek SK., 2022). Given the well-documented genetic variability of IBDV, monitoring the genetic evolution and virulence shifts in circulating strains is crucial to mitigating the economic consequences of vvIBDV. This study aimed to detect IBDV in the bursa of Fabricius, perform molecular characterization and phylogenetic analysis of the identified strains. By examining both the VP1 and VP2 genes, we sought to enhance the understanding of IBDV evolution in Türkiye.

## MATERIALS AND METHODS

### Study population and sampling

Commercial chicken flocks (broilers and layers) were selected from from five geographic regions of Türkiye: the Aegean, Marmara, Black Sea, Eastern Anatolia, and Mediterranean and sampled from January 2024 to March 2025. All broiler flocks had been vaccinated using only hatchery-administered vaccines (Immune complex vaccine/Winterfield 2512 strain or Vector vaccine) while all layers had farm-administered vaccines (Winterfield 2512, GM97, D78/CH80 or MB strain) at 15–29 days of age and some had hatchery-administered vaccines (Immune complex vaccine/SYZ26 strain or Vector vaccine). Sampling aimed primarily to monitor vaccine uptake and to diagnose birds suspected of IBDV infection. For this, two criteria were considered to select flocks for sampling: 1. Flocks suspected to have subclinical and clinical IBD; 2. Randomly selected flocks without IBD signs for vaccine uptake. From each broiler flock, 5 birds at the age of 19 to 41 days were humanely euthanized and gross lesions were recorded. For layers 5 to 10 samples were taken from each flock at the age of 26 to 51 days. A total of 1554 bursa of Fabricius samples representing 280 flocks of broilers and 23 flocks of layers were collected for molecular virology (Table 1). Sample IDs and information about sampling area have been summarized in supplementary data set (Supplementary Table 1). In order to investigate potential reassortant IBDVs in archived very virulent IBDV viruses (2020-2023), 16 Bursa of Fabricius specimens were also analyzed by RT-PCR. In this study, samples were collected by the registered Veterinarians. The informed consent was obtained from farm owners. During the collection of samples, all applicable national, international, and/or institutional guidelines for the care and use of animals were followed.

**Table 1:** Summary of IBDV detection (based on VP2 sequence).

Production type	Number of Flocks	Total samples	Positive samples	Negative samples	Total sequenced	vvIBDV Strains	Other Strains	Sequencing results			Age	Statistical analysis
Broilers	280	1400	1225	175	1225	549	676	Strain		NO	19-41 days	Prevalence (broiler vs. layer) χ²= 0.865 p = 0.352 Strain Distribution χ²= 10.21 p = 0.0014
								ICX/ W2512		640		
								Faragher		36		
								MB		0		
								Lukert		0		
								D78		0		
								vvIBDV		549		
Layers	23	154	139	15	139	41	98	Strain	NO		26-51 days	
								D78	16			
								Faragher	8			
								228E/SYZ 26	13			
								Lukert	0			
								MB	53			
								W2512	8			
								vvIBDV	41			
Total	303	1554	1364	190	1364	590	774	-	1364		-	-

### RNA extraction and reverse transcription

The bursa of Fabricius samples were thawed, homogenized, and processed for RNA extraction using a commercial nucleic acid extraction kit as described by the manufacturer (innuPREP Virus DNA/RNA Kit, Catalog No: KS 20800250, Analytik Jena, Berlin, Germany). The RNA concentration was quantified spectrophotometrically (NanoDrop 1000c, Thermo Scientific, Waltham, MA, USA). Subsequently, cDNA was synthesized from 50 μL of extracted RNA using the High-Capacity cDNA Reverse Transcription Kit (Thermo Fisher Scientific, Cat. No. 4368814). For each reaction, 10 μL of 2X RT master mix was combined with 10 μL of RNA in a 96-well plate or individual tube. The mixture was gently pipetted to homogenize, briefly centrifuged to remove air bubbles, and kept on ice until thermal cycling. Reverse transcription was performed as recommended by the manufacturer.

### Sequencing of Partial VP1 and VP2 genes

In order to differentiate between vaccine and field strains of IBDV all samples were analysed by RT-PCR for IBDV-VP2 gene. Field and vaccine strains were confirmed by partial VP2 gene sequencing. For VP2 gene sequencing, RT-PCR was performed using IBDV-specific primers (forward: 5-CGCTATAGGGCTTGACCCAAAAA-3 and reverse:

5-CTCACCCCAGCGACCGTAACGACG-3) as previously described (Toroghi et al., 2001) The assay amplified a 552 bp fragment (nucleotide positions 651–1202) of the VP2 gene. The PCR reaction mixture (50 μl total volume) consisted of 3 μl (10 μM) each of forward and reverse primers, 25 μl Maxima Hot Start PCR Master Mix (Thermo Scientific, K1052), 15 μl nuclease-free water, 1 μl MgCl₂ (25 mM), and 3 μl cDNA. Amplification was carried out in a thermal cycler (Bio-Rad, Chromo-4) under the following conditions: initial denaturation at 95°C for 10 min, followed by 35 cycles of 94°C for 1 min, 60°C for 1 min, and 72°C for 1 min, with a final extension at 72°C for 5 min.

Samples that showed a strong positive result (a bright band) on gel electrophoresis for RT-PCR were further analyzed with a more precise and quantitative technique (probe-based real-time RT-PCR) to measure viral load (Ct) in original samples. The primers, PCR protocol and conditions were applied as reported previously (Iván et al., 2005) using a real time PCR instrument (Thermo Fisher Scientific, Applied biosystems, StepOnePlus). Very virulent IBDVs having Ct value lower than 30 and confirmed by VP2 sequencing were further anlaysed by sequencing for partial VP1 gene. For VP1 gene sequencing, a 716 bp fragment of the VP1 gene was amplified using primers B293U (5′-TTTTGCAGCCGCGGTCTCT-3′) and B1008L (5′-GTTTGACCCCTTTGTCCCTGC-3′). The PCR protocol included: (1) initial denaturation at 95°C for 5 min, (2) 35 cycles of 95°C for 30 s, 56°C for 30 s, and 72°C for 1 min, and (3) a final elongation at 72°C for 10 min (Alfonso-Morales et al., 2015;Jiang et al., 2021). A no-template control (nuclease-free water) and positive control (consisting of previous IBDV-confirmed samples) were included in all PCR reactions. PCR products were visualized using 1.5% agarose gel (Invitrogen™ UltraPure™ Agarose, Cat. No. 11553277) and subsequently purified. The amplicons from positive samples were submitted for Sanger sequencing to a commercial laboratory (MedSanTek, Istanbul, Türkiye). The obtained nucleotide sequences from both genes were submitted to the GenBank database via the Bankit submission tool (NCBI; http://www.ncbi.nlm.nih.gov/Genbank) (PV557458-PV557492 for VP1 and PV557493-PV557527 for VP2) .

### Phylogenetic and evolutionary analyses

To assess the genetic relatedness of wild-type IBDV strains, nucleotide BLAST analysis was performed against reference sequences. Sequence identity and similarity were calculated using the Sequence Identity and Similarity (SIAS) tool (Complutense University of Madrid). Sequence alignment and editing were conducted in BioEdit (Hall, 1999), with reference sequences trimmed to match the amplified regions of the VP1 and VP2 genes. Wild-type sequences with low coverage, resulting in truncated alignments, were excluded from further analysis. Phylogenetic relationships were reconstructed using the Neighbor-Joining method in MEGA software (Tamura et al., 2021), with bootstrap support calculated from 1,000 replicates. To characterize the molecular properties of the studied IBDV strains, the nucleotide and deduced amino acid sequences of both genes were analyzed alongside reference IBDV strains from NCBI, including various vaccine strains used in Türkiye The analysis incorporated sequences from Nobilis® Gumboro D78 (D78 strain), Nobilis® Gumboro 228E (228E strain), CEVAC® IBD L (Winterfield-2512 strain), Bursine®-2 (Lukert strain), HVT vaccine (Faragher 52/70 strain) and Bursine® Plus (Bursine 2 strain) for both the hvVP2 and VP1 genes.

### Aminoacid analyses

Comparative amino acid sequence analysis of both VP2 and VP1 proteins was conducted against reference strains using BioEdit software.

### Recombination analysis

Potential recombination events were investigated using RDP5 (Martin et al., 2021) which implements seven detection algorithms: RDP, GENECONV, 3Seq, Chimera, Bootscan, SiScan, and MaxChi.

**Statistical analysis:** Statistical analyses were performed using R software (version 3.1.0; R Foundation for Statistical Computing, Vienna, Austria). Differences in IBD prevalence were assessed using Chi-squared (χ²) tests, with a significance level set at P < 0.05.

## RESULTS

### Clinical and gross findings

**Fig. 1:**
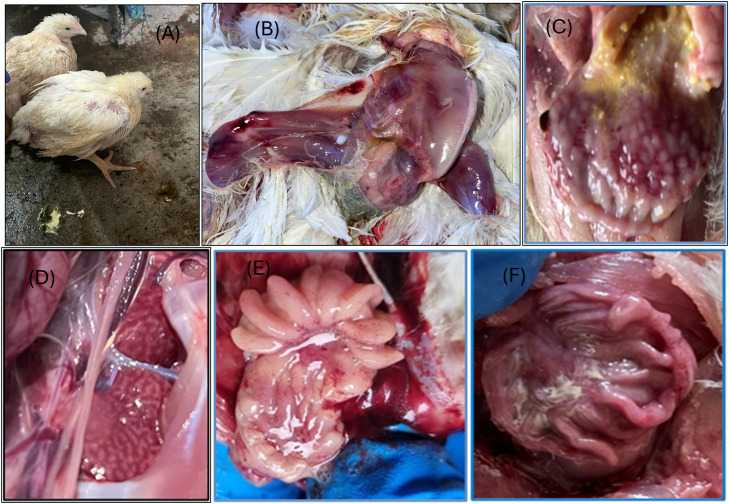
Clinical signs and necropsy lesions. (A) Dull and anorexic chicks with whitish watery diarrhea. (B) Carcass showing hemorrhages on breast and thigh muscles. (C) hemorrhages on proventriculus (glandular stomach). (D) Petechial hemorrhages on Kidney (E) Hemorrhages on Bursa of fabricius (F) yellowish cheesy material on Bursa of Fabricius.

Infected birds exhibited characteristic clinical signs, including depression, ruffled feathers, lethargy, reduced feed intake, stunted growth, and white watery diarrhea (Figure 1). Additionally, fecal staining was observed around the cloacal feathers. In clinically affected chicken flocks, mortality rates ranged from 9 % to 16 %. At necropsy, gross pathological changes were observed in the bursa of Fabricius across chicken flocks infected with IBDV. Notable findings included severe atrophy, with some bursae becoming firm and shrinking to approximately one-third of their original size. Other lesions included distinct edema, hemorrhage, atrophy, and occasional enlargement of the bursae. Additionally, petechial and linear hemorrhages were evident on the pectoral mscles, leg muscles, proventriculus (glandular stomach), kidney, and bursa of fabricius of some affected birds (Figure 1).

### Frequency of IBDV vaccines and field strains

Among 1,400 bursa of Fabricius samples collected from broilers, 1,225 (87.5%) tested positive for IBDV by RT-PCR, displaying a 552 bp amplicon corresponding to the partial VP2 gene upon gel electrophoresis. Similarly, 139 of 154 (90.2%) samples from layers were positive for the IBDV-VP2 gene (Table 1). There was no statistically significant difference in IBDV prevalence (i.e., the proportion of positive samples) between broiler and layer flocks. The slight difference observed in the data (87.5% vs 90.2%) is very likely due to random chance. vvIBDV was detected in 549 (549/1225, 44.8%) broiler samples and 41 (41/139, 29.4%) layer samples. Broilers had a significantly higher prevalence of vvIBDV than layers (p=0.0014).

Vaccine strains identified in broilers included the Immune complex vaccine/W2512 (n=640) and Faragher (n=36). In layers, the following strains were detected: W2512 (n=8), Faragher (n=8), 228E/SY (n=13), D78 (n=16), and MB (n=53). Additionally, 35 VP2-positive samples were analyzed for the VP1 gene, revealing a 716 bp amplicon (Table 1). A total of 33 samples from broiler flocks and 2 samples from layer flocks were sequenced (Supplementary Table 1).

Among the 16 archived Bursa of Fabricius samples (2020–2023), eight were confirmed via RT-PCR and produced high-quality bands and sequences for analysis. The VP2 gene accessions are PV637746-PV637752 and PV661754, while the VP1 gene accessions are PV637739-PV637745 and PV661753.

**Fig. 2:**
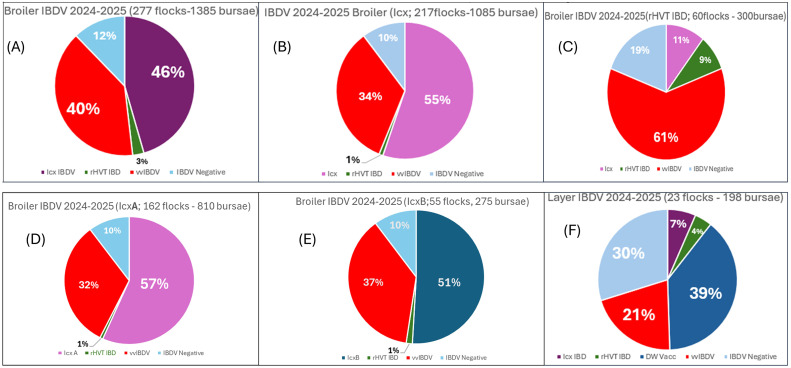
(A) IBDV detection in broilers vaccinated with different type of vaccine. (B) Detection of IBDV in broilers vaccinated with Immune complex IBDV vaccine (Icx) (C) IBDV detection in broilers vaccinated with vector IBDV vaccine (rHVT IBD). (D) IBDV detection in broilers vaccinated with Immune complex IBDV vaccine (Icx-A). (E) IBDV detection in broilers vaccinated with Immune complex IBDV vaccine (Icx-B). (F) IBDV detection in layers vaccinated with different type of vaccine.

The Figure 2 presents the detection of IBDV in broilers and layers vaccinated with different vaccine types. In broilers (277 flocks, 1385 bursae), varying proportions of vvIBDV strains were observed. Among broilers vaccinated with immune complex (Icx) and recombinant (rHVT-IBD) vaccines, vvIBDV detection rates were 34% and 61%, respectively. Similarly, in broilers vaccinated with Icx-A and Icx-B (Gmbhtc), vvIBDV was detected at 32% and 37%, respectively. In layers (23 flocks, 198 bursae), a mix of recombinant, and wild-type strains was observed, with wIBDV detected in 39% of samples. The findings highlight the persistence of field strains despite vaccination, with varying efficacy across vaccine types.

### Phylogenetic analysis of the partial VP2

**Fig. 3:**
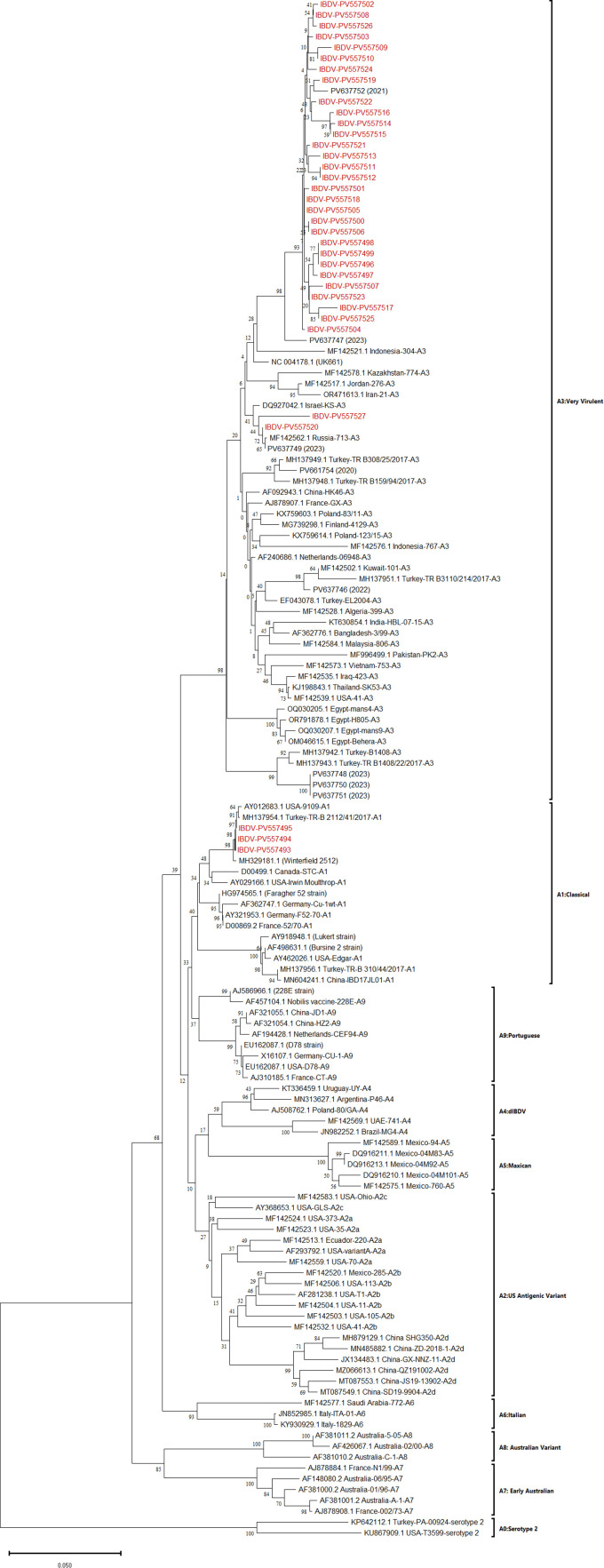
Phylogenetic tree based on partial sequences of VP2 gene. Evolutionary history was inferred using the Neighbor-Joining method. The percentage of replicate trees in which the associated taxa clustered together in the bootstrap test (1000 replicates) are shown next to the branches. The evolutionary distances were computed using the p-distance method and are in the units of the number of base differences per site. This analysis involved 142 nucleotide sequences. Evolutionary analyses were conducted in MEGA11. IBDV strains in study were represented with red color.

Phylogenetic analysis of partial VP2 gene sequences revealed that the IBDV strains in this study segregated into two distinct genogroups: A1 and A3. Furthermore, serotype II IBDV strains formed a distinct clade, clearly separated from all serotype I strains. Among these, 32 strains clustered within genogroup A3, which is typically associated with very virulent (vv) IBDV strains, while three strains grouped into genogroup A1, comprising classical virulent or vaccine-derived strains. Notably, three strains (PV557493, PV557494, and PV557495) exhibited close evolutionary proximity to MH137954 (a Turkish strain from 2017) and MH329181 (Winterfield 2552), suggesting either shared vaccine origins or recent common ancestry among circulating strains. Ancestral strains were positioned near the root of the phylogenetic tree, potentially representing older lineages or historical interspecies transmission events. Genogroup A1 included most reference vaccine strains (e.g., D78, Winterfield, Faragher) and was characterized by short branch lengths, indicating high genetic conservation and likely reflecting widely distributed vaccine-derived lineages. In contrast, genogroup A3 displayed longer branch lengths, suggesting ongoing evolutionary diversification. Within this genogroup, two strains (PV557527 and PV557520) formed a distinct subcluster, showing genetic relatedness to IBDV strains from Israel (DQ927042) and Russia (MF142562). Additionally, 30 strains from this study formed a separate cluster, implying a common ancestral origin (Figure 3).

### Phylogenetic analysis of the partial VP1

**Fig. 4:**
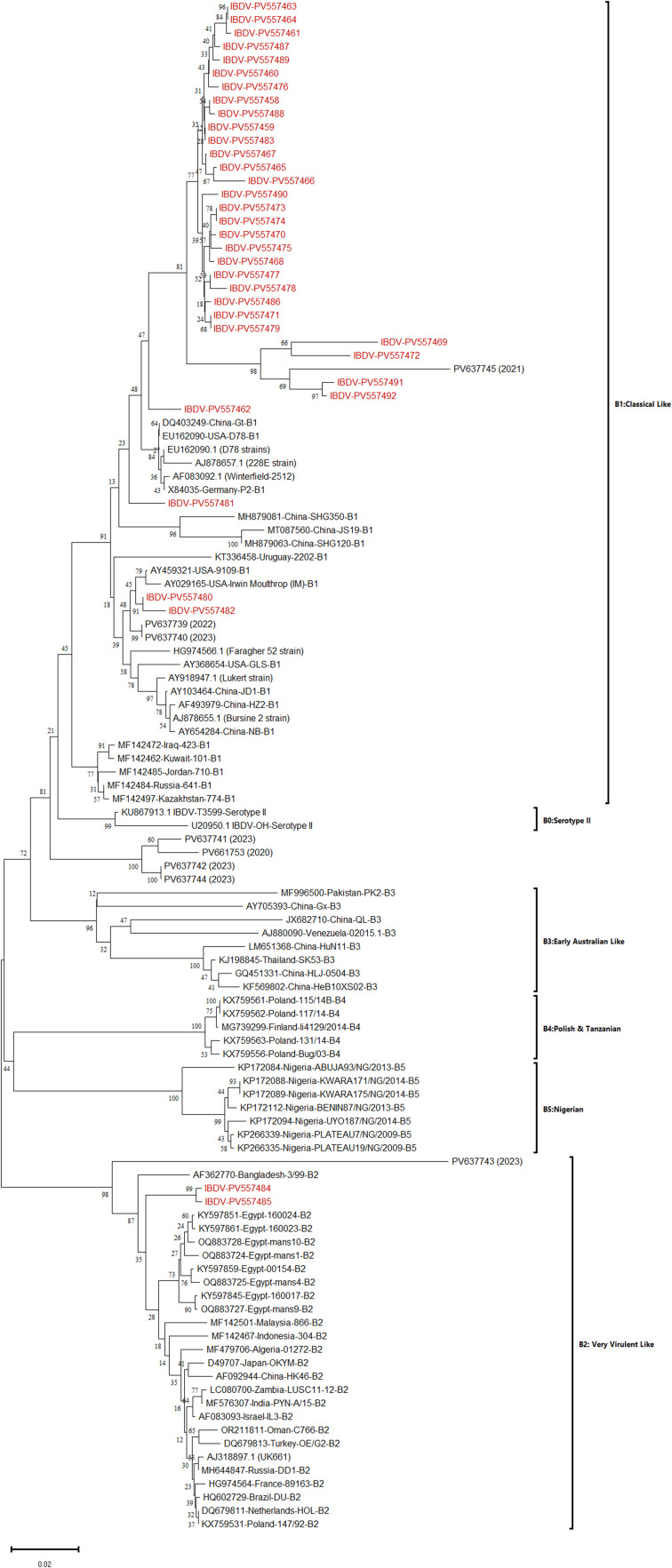
Phylogenetic analysis of IBDV strains based on partial VP1 gene sequences. The evolutionary history was reconstructed using the Neighbor-Joining method (MEGA11), with bootstrap values (1000 replicates). Evolutionary distances were calculated using the p-distance method (units: base differences per site). The tree includes 114 nucleotide sequences, with study strains highlighted in red. Reference lineages (e.g., classical, very virulent, and reassortant genotypes) are labeled for context. Scale bar indicates genetic divergence.

Very virulent IBDV isolates exhibiting a high viral load (Ct < 30, as determined by probe-based real-time RT-PCR) were subjected to partial VP1 gene sequencing and phylogenetic analysis. The phylogenetic tree constructed from the partial VP1 gene segregated the IBDV strains into two major genogroups: B1 (classical/vaccine strains) and B2 (vvIBDV). Among the 35 analyzed sequences, 33 clustered within genogroup B1, while two strains (PV557584, PV557585) grouped into genogroup B2, confirming their very virulent nature. Two strains (PV557480, PV557482) formed a subcluster closely related to the reference strain AY029165 (Irwin Moulthrop). Four strains (PV557469, PV557472, PV557491, PV557492) clustered separately within the B1 genogroup, consistent with classical IBDV. Only two strains (PV557584, PV557585) were confirmed as highly pathogenic (genogroup B2) (Figure 4).

Combining the characteristics of the representative regions of segments A and B, the 35 strains detected in this study could be divided into the following three genotypes: A3B1(30/35), A1B1 (3/35) and A3B2 (2/35). The reassortants (A3B1) derived segment A from vvIBDV (genogroup A3) and segment B from attenuated/non-virulent strains (genogroup B1). Additionally, serotype II strains formed a distinct phylogenetic clade, clearly divergent from all serotype I strains.

### Nucleotide analysis of VP2 gene

Nucleotide sequence analysis revealed substantial genetic variation among the studied IBDV strains, with VP2 gene identities ranging from 90.23% to 99.57%. Comparative analysis with reference strains including the field strain UK661 and vaccine strains (D78, 228E, Winterfield 2512, Lukert, Faragher 52/70, and Bursine 2) revealed a broader nucleotide homology range of 89.38% to 99.57% (Supplementary Table 2). Strains IBDV-PV557493, IBDV-PV557494, and IBDV-PV557495 showed near-complete identity (99.57%) with MH329181 (Winterfield 2512). The greatest divergence was observed between IBDV-PV557517 and AY918948.1 (Lukert strain). Multiple alignment analysis identified non-synonymous substitutions in hypervariable regions of VP2 but no insertions or deletions across the VP2 gene.

### Nucleotide analysis of VP1 gene

Nucleotide identity among the VP1 sequences of the IBDV strains in this study ranged from 84.02% to 99.65%. Comparative analysis of the VP1 gene between the 35 studied strains and reference strains including the field strain (UK661) and vaccine strains (D78, 228E, Winterfield-2512, Lukert, Faragher 52/70, and Bursine 2) revealed nucleotide identities ranging from 73.43% to 98.61% (Supplementary Table 3). The highest identity (98.61%) was observed between IBDV-PV557480 and Faragher 52 (HG974566.1), while the lowest (73.43%) was between IBDV-PV557484, IBDV-PV557485, and the 228E strain (AJ878657.1).Notably, IBDV-PV557480 exhibited exceptional similarity to multiple reference strains, particularly Faragher 52 (98.61%), whereas 228E and Lukert displayed the lowest similarity (73.43%–73.61%) with PV557484 and PV557485. Most strains maintained high similarity (>90%) with D78, Winterfield-2512, Bursine 2, and Faragher 52. Interestingly, the UK661 reference showed moderate similarity (high 80s%) with most strains, except for PV557484 and PV557485, which exhibited unusually high identity (97.39% and 97.74%, respectively).

### Deduced amino acid analysis of VP2 protein

To facilitate a comprehensive comparative analysis, we sequenced the hypervariable region (HVR) of the VP2 gene from a set of IBDV strains (PV557493-PV557527) in this study. The deduced amino acid sequences were subsequently aligned with the reference strain UK661 (NC_004178). The amino acid identity between the VP2 proteins of the IBDV strains ranged from 91.08% to 100%, while comparison with the reference strain revealed identities ranging from 87.26% to 100% (Supplementary Table 4). Three strains (PV557493, PV557494, PV557495) exhibited complete identity (100%) with the Winterfield 2512 strain (MH329181.1), indicating near-identical genetic sequences. In contrast, two strains (PV557509, PV557517) demonstrated the lowest identity (87.26%) with the Lukert strain (AY918948.1), suggesting notable genetic divergence from this reference.

**Fig. 5:**
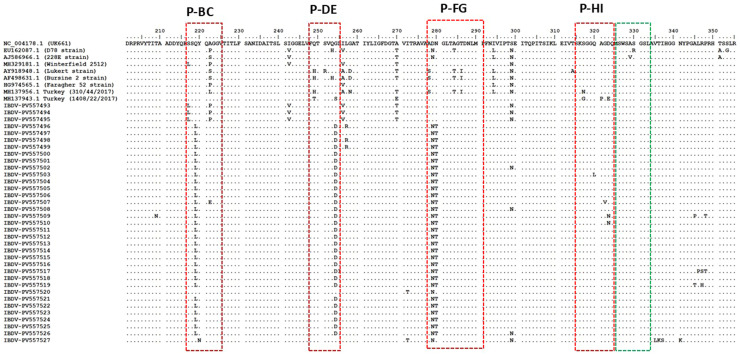
Alignment of the HVR amino acid sequences of VP2 gene (segment A) The hypervariable region (HVR) of the VP2 gene from the studied IBDV strains (PV557493-PV557527) was sequenced and the amino acid sequence was deduced. These sequences were aligned with reference very virulent (vvIBDV) and non-vvIBDV strains to for comparative analysis. The alignment was compared to the reference strain UK661 (Accession number NC_004178), where identical amino acids are denoted by a dot. The major hydrophilic peaks, corresponding to the P-BC, P-DE, P-FG, and P-HI loops, are highlighted with “red” boxes while the serine‑rich heptapeptide (326-332aa: SWSASGS), a known molecular marker of vvIBDVs has been highlighted with “green”box.

The comparative analysis also revealed several key amino acid substitutions in the VP2 protein of the study strains, relative to the reference strains (Supplementary Table 4). Notably, all aligned sequences contained the serine-rich heptapeptide (positions 326-332, SWSASGS), a well-characterized molecular marker associated with vvIBDVs (green box, Figure 5).Furthermore, antigenic drift was evident in the HVR of VP2, particularly within four hydrophilic loops PBC, PDE, PFG, and PHI (**highlighted in red boxes in** Figure 5). These loops are critically involved in IBDV virulence and in determining the virus’s antibody neutralization profile, underlining their importance in both immunological and pathogenic characteristics of IBDV.

Regarding genogroup-specific features, IBDV strains within genogroup A3 displayed a distinct pattern of amino acid substitutions (222A, 242I, 253Q, 256I, 279D, 284A, 294I, and 299S), consistent with the profile of vvIBDV strains. In contrast, the strains classified under genogroup A1 (PV557493-PV557495) exhibited a classical strain amino acid profile (222P, 249Q, and 286T), distinguishing them from more virulent strains. An interesting mutation at position N299 was observed in several of the study strains (PV557493, PV557494, PV557495, PV557503, PV557509, PV557526, PV557527), which was absent in the reference strain UK661 (NC_004178). This mutation may hold potential significance for further investigation into the genetic variability and evolution of IBDV

### Deduced amino acid analysis of VP1 protein

We sequenced the partial VP1 gene from a set of IBDV strains (PV557458-PV557492) in this study and aligned the resulting deduced amino acid sequences with the reference strain UK661 (Accession AJ318897.1) for comparative analysis. The amino acid identity among the VP1 sequences of the study strains ranged from 86.45% to 100%. When compared to the reference strains, the identity ranged from 86.45% to 99.47% (Supplementary Table 5).

Several strains (PV557458-460, PV557463-464, PV557467, PV557470-471, PV557475, PV557477, PV557479-481, PV557483, PV557486, PV557490) exhibited 99.47% similarity with both the D78 and Winterfield 2512 reference strains. The lowest identity (86.45%) was observed between strain PV557469 and the UK661 strain (AJ318897.1). Interestingly, strains PV557484 and PV557485 displayed the highest similarity to UK661, rather than to D78 or Winterfield.

**Fig. 6:**
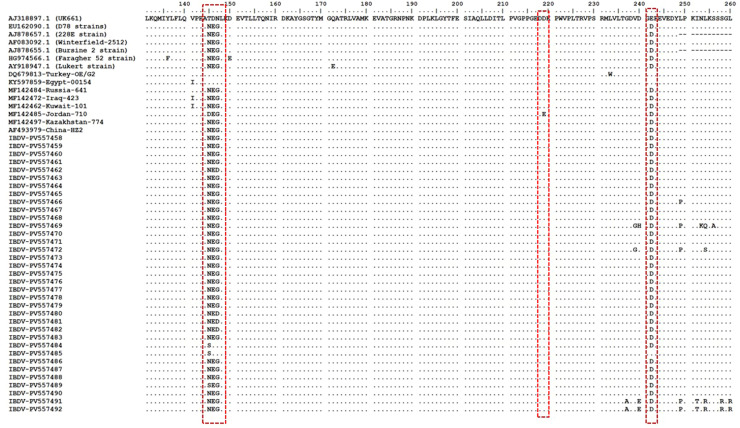
Alignment of the partial VP1 amino acid sequences (segment B). The deduced amino acid sequences of the VP1 gene from the IBDV strains in this study (PV557458-PV557492) were aligned with reference vvIBDV and non-vvIBDV strains to identify molecular signatures. The alignment was performed against the UK661 strain (Accession AJ318897.1), where identical residues are represented by dots.

A key finding in this analysis is the absence of the characteristic TDN tripeptide (positions 145-147, TDN), a known marker of very virulent IBDV (vvIBDV). Instead, 33 of the sequences contained the NEG marker, while two sequences harbored the SEG marker at the same position. Both of these markers are indicative of non-vvIBDV strains belonging to genogroup B1 (Figure 6).

### Recombination Analysis

Recombination analysis of the VP2 gene showed no evidence of recombination events in the IBDV strains examined in this study. However, indications of recombination were detected in the VP1 gene sequences of a few isolates. For instance, strain PV557458 appeared to have PV557460 as a potential major parent and PV557459 as a minor parent, while strain PV557485 likely originated from PV557461 (major parent) and PV557472 (minor parent).

## DISCUSSION

Poultry diseases pose a major threat to the global poultry industry, resulting in substantial economic losses due to high mortality rates, reduced productivity, trade restrictions, and elevated costs for disease prevention and treatment (Müller et al., 2012;Alkie and Rautenschlein, 2016;Pikuła et al., 2020;Zhang et al., 2022). Since the initial detection of the vvIBDV in Europe in 1980, Türkiye has experienced multiple outbreaks, inflicting severe economic damage on its poultry sector. Although widespread vaccination and improved management practices have helped mitigate vvIBDV cases, the emergence of novel variants and reassortants in immunized flocks has become a growing concern. These strains provoke acute bursal lesions targeting the chicken’s central immune organ leading to severe immunosuppression and significant weight loss in affected birds. Moreover, the resulting immunosuppression compromises the efficacy of concurrent vaccinations against critical pathogens such as avian influenza and Newcastle disease, presenting a major challenge to poultry disease control strategies (Yilmaz et al., 2029; Kurtbeyoğlu and Akan, 2024).

As part of ongoing surveillance of IBDV field strains and monitoring vaccine strains in Türkiye, we analyzed 1554 bursal samples collected from 303 chicken farms in 2024 and 2025 using RT-PCR for sequencing to investigate the epidemiology and genetic diversity of IBDV strains circulating in Turkish flocks. During this study, we sequenced the HVR of the VP2 gene and a partial segment of the VP1 gene. The obtained sequences were then aligned and analyzed through phylogenetic reconstruction to assess genetic relationships and evolutionary trends among the detected strains. Overall detection rate for IBDV by RT-PCR was 87.77% (1364/1554) of bursa Fabricius samples) in this study. The phylogenetic analyses of VP1 and VP2 sequences revelaed that IBDV strains detected in this study were divided into the following three genotypes: A3B1 (30/35), A1B1 (3/35) and A1B2 (2/35).

Reassortant IBDV strains of genogroup A3B1 were identified as the predominant circulating strains (30/35, 85.7%) among Turkish poultry flocks in the present study. This finding contrasts with reports from Mossad et al (2024). and Yilmaz et al (2019) who reported the predominance of A3B2 and classical strains in Egypt and Türkiye respectively.

In Europe, reassortant viruses of genogroup A3B1 were found to be widespread in 2020, detected in Latvia, Germany, Belgium, Denmark, the Netherlands, the Czech Republic, and Sweden (Mató et al., 2020). Some of these strains carried key mutations (Q219L, G254D, D279N, and N280T) in HVR, indicating ongoing evolution through antigenic drift. By 2023, the presence of A3B1 strains had expanded further, with reports from France, Italy, Portugal, Spain, and the UK (Legnardi et al., 2022), confirming a broader geographical distribution than previously recognized. The genome of IBDV consists of two double-stranded RNA segments, and segment reassortment serves as a key evolutionary mechanism driving viral diversity. When IBDV strains of different genotypes co-infect a host, random exchange of genome segments can generate novel genotypes (Wei et al., 2008;Jiang et al., 2021). Co-infection has long been recognized as a prerequisite for segment exchange between different IBDV genotypes. Recent studies confirm that when two genotypes co-infect the same cell, their viral replication factories co-localize, facilitating segment reassortment (Wei et al., 2008;Campbell et al., 2020). Additionally, wild birds may act as reservoirs for IBDV, introducing the virus to poultry populations and increasing the risk of co-infection with multiple genotypes a potential driver of reassortment (Naggar et al., 2020;Orakpoghenor et al., 2020b).

IBDV reassortment events have been increasingly reported worldwide. The first documented case occurred in 2003 in China when researchers identified a novel reassortant strain containing a very virulent (vv) segment A paired with a non-vv segment B (Sun et al., 2003). This finding was further supported by Nouën et al. (Nouën et al., 2006), who analyzed 50 historical strains (1972-2002) from 17 countries across four continents. Their study revealed phylogenetic incongruencies between segments A and B in 26% of strains, with sequencing confirmation showing a vv segment A and non-vv segment B in at least one case. Subsequent reports have documented similar reassortant strains with this characteristic genomic configuration in multiple countries. For example, reassortant IBDV strains of genogroup A3B1 have previously been detected in China (Gao et al., 2007;Jiang et al., 2021), India (Raja et al., 2016), South Korea (Lee et al., 2017), Venezuela (Nouën et al., 2006), Brazil (Fernandes et al., 2012), Ethiopia (Jenberie et al., 2014), Nigeria (Nwagbo et al., 2016), Algeria (Abed et al., 2018), Zambia (Kasanga et al., 2013), UK (Reddy et al., 2024), Europe (Pikuła et al., 2018;Mató et al., 2020;Pikuła et al., 2020;Legnardi et al., 2022;Legnardi et al., 2023), Türkiye (Kurtbeyoğlu and Akan, 2024), and the Near East and Persian Gulf regions (Legnardi et al., 2024).

While genogroup A3B1 remains predominant, novel reassortant genotypes have emerged, including A3B3 in Bangladesh (Nooruzzaman et al., 2022), A1B2 in Egypt (Mosad et al., 2024), A3B4 in Poland (Pikuła et al., 2023), and A3B5 in Nigeria (Nwagbo et al., 2023).

Furthermore, some IBDV strains have been reported to exhibit unconventional segment pairings, including non-vv segment A with vv segment B in China (Wei et al., 2006;Cui et al., 2013). Rare inter-serotype reassortants (vv segment A + serotype 2 segment B) in Europe (Soubies et al., 2017) and the US (Stoute et al., 2019) have also been reported. Notably, only two very virulent (vv) strains of genogroup A3B2 were detected in our surveillance (2/35, 5.7%). This markedly low prevalence compared to A3B1 reassortants (85.7%) suggests a potential competitive advantage of A3B1 strains, possibly through enhanced fitness or transmission efficiency. However, comprehensive comparative studies of viral fitness parameters are needed to substantiate this competitive exclusion hypothesis (Reddy et al., 2024). These findings highlight the increasing genetic complexity of circulating IBDV strains through diverse reassortment patterns.

In the present study, the hvVP2-deduced amino acid sequences have vvIBD amino acid signature with the characteristic serine-rich heptapeptide (326-332aa) sequence SWSASGS of vvIBDVs (Mosad et al., 2024). The virulence hallmark amino acids (222A, 242I, 249Q, 253Q, S254, 256I, G258, T260, T269, A270, 272I, A278, 279D, 284A, 286T, 294I, 299S, 318G, A321, 324L, and S330) were identified in our five vvIBDV strains. These hallmark amino acids are thought to be responsible for the antigenicity and virulence of IBDV (Jackwood et al., 2018;Pikuła et al., 2018;Mosad et al., 2024). Comparative analysis revealed distinct amino substitution patterns (compared to UK661). The surface P-BC loop (Y220) remained conserved across most Turkish strains, except PV557527 (Y220N). Notable substitutions occurred in P-DE loop (G254D) and P-FG loop (D279N, N280T). Attenuated strains showed characteristic substitutions (S217L, A222P, I242V, I256V, A270T, N293L, S299N) distinguishing them from virulent field strains. Our analysis identified critical amino acid substitutions (Q219L, G254D, D279N, N280T, T250S, and S251I) localized within three of the four major hydrophilic loops comprising the HVR apex, which is a key antigenic domain responsible for neutralizing antibody binding (Alkie and Rautenschlein, 2016). These mutations occur in structurally critical positions. The substitutions alter surface-exposed residues in the immunodominant PBC, PDE, and PFG loops. The observed substitutions may enable viral escape through (1) steric hindrance of antibody binding and (2) disruption of conserved conformational epitopes. Such mutations could compromise vaccine-induced immunity, potentially explaining field reports of vaccination breakthroughs (Reddy et al., 2024). The accumulation of multiple substitutions in this immunodominant region suggests ongoing antigenic drift, warranting surveillance of their epidemiological impact on vaccine efficacy.

Notably, our analysis revealed that all VP1 sequences in this study lacked the TDN tripeptide (positions 145-147aa), which serves as a molecular hallmark of vvIBDV strains (Jackwood et al., 2018;Pikuła et al., 2020;Mosad et al., 2024). Instead, we observed two distinct markers associated with genogroup B1 strains. The majority of sequences (33/35) contained the NEG marker. A minority (2/35) exhibited the SEG marker at the same position. These findings provide clear molecular evidence that the circulating strains in our study belong to the non-vvIBDV (genogroup B1) classification. The consistent absence of the TDN motif, coupled with the presence of alternative markers (NEG/SEG), strongly supports this genotypic characterization. We also observed one additional amino acid substitution (E242D) in non-vvIBD (genogroup B1) when compared to UK661 strain. The recombination analysis revealed no detectable recombination signals in the VP2 gene. However, evidence of recombination was observed in the VP1 gene of certain strains. Homologous recombination plays a key role in driving antigenic variation and altering viral pathogenicity. Previous studies have reported that recombination events among classic, very virulent, attenuated, and variant IBDV strains in VP2 can lead to the exchange of antigenic epitopes and modifications in pathogenicity (Huang et al., 2022).

Our study had a few limitations: (1) In this study, only VP1 and VP2 genes of IBDV were analysed. Reassortant strains have been found to harbor mutations in other genomic regions, including those encoding VP3, VP4, and VP5 (Mato et al ., 2020) . Thus, expanding sequencing efforts to cover the other genes would help determine whether similar mutations exist in Turkish strains. (2) We did not conduct *in vivo* experiments in chickens to assess the pathogenicity of the reassortant strains. The immunological and evolutionary consequences of co-infection involving reassortant A3B1 strains alongside classical or vaccine IBDV strains remain poorly understood. This represents a critical knowledge gap, as such interactions could significantly influence viral fitness, transmission dynamics, and pathogenicity.

## CONCLUSIONS

This study revealed that the reassortant genogroup A3B1 was the predominant strain in Turkish chicken flocks, followed by classical IBDV (A1B1) and very virulent IBDV (A3B2). This study represents the first report documenting the predominance of the A3B1 genotype in Türkiye. Additionally, we provide evidence that segment reassortment among circulating IBDV strains has contributed to the emergence of novel genotypes in the region. However, key aspects of A3B1, including its pathogenesis, clinical manifestations, immunosuppressive effects, and potential for immune evasion, remain poorly characterized. Further comprehensive studies are essential to elucidate the viral fitness and pathogenicity of this reassortant strain, which will be critical for developing effective preventive strategies against IBDV.

## SUPPLEMENTARY DATA

Additional supporting data, including supplementary tables and figures, are provided in the supplementary materials.

## DATA AVAILABILITY STATAMENT

All data are included in the manuscript. The data is available up on request from the corresponding author. All the sequences generated have been deposited in NCBI GenBank database with accession numbers PV557458-PV557527. Sequences submitted to GenBank can be downloded from NCBI (https://www.ncbi.nlm.nih.gov/nuccore). The datasets generated during and/or analyzed during the current study are available from the corresponding author upon request.

## CRediT authorship contribution statement

**Erhan Bayraktar:** Investigation. **Ozge Aydin:** Investigation, Formal analysis. **Hasan Emre Tali:** Investigation, Formal analysis. **Ismail Egemen Ozkan:** Investigation, Formal analysis. **Sajid Umar:** Writing – review & editing, Writing – original draft, Visualization, Software, Data curation. **Hamid Besim Tali:** Investigation. **Aysun Yilmaz:** Writing – review & editing, Supervision, Project administration, Methodology, Funding acquisition, Formal analysis, Data curation, Conceptualization. **Nuri Turan:** Validation, Resources, Project administration, Methodology, Conceptualization. **Ahmet Kolukisa:** Methodology, Investigation. **Cem Konuk:** Investigation. **Altug Erdem:** Investigation. **Abubakar Siddique:** Writing – review & editing, Data curation. **Munir Iqbal:** Writing – review & editing, Software, Resources. **Juergen A. Richt:** Writing – review & editing, Software, Resources, Funding acquisition. **Huseyin Yilmaz:** Writing – review & editing, Validation, Supervision, Resources, Project administration, Funding acquisition, Formal analysis, Data curation, Conceptualization.

## Disclosures

There is no conflict of interest among authors of this manuscript.

## Appendix Supplementary materials

Image, application 1

Image, application 2

Image, application 3

Image, application 4

Image, application 5

## Footnotes

Supplementary material associated with this article can be found, in the online version, at doi:10.1016/j.psj.2025.105974.
