# Supplementary material for: Predominance of reassortant infectious bursal disease viruses in Turkish poultry flocks
Source: Poult Sci. 2025 Oct 20;104(12):105974. doi: 10.1016/j.psj.2025.105974 (PMC12747199; doi:10.1016/j.psj.2025.105974)
Supplement: Supplementary file 1 [file mmc1.docx]

Supplementary Table 2: Nucleotide homology analysis of the VP2 gene among the 35 IBDV strains in this study and reference vaccine strains

| **IBDV strains** | **NC_004178.1**  **(UK661)** | **EU162087.1**  **(D78)** | **AJ586966.1**  **(228E)** | **MH329181.1**  **(Winterfield 2512)** | **AY918948.1**  (Lukert) | **AF498631.1**  (Bursine 2) | **HG974565.1**  **(Faragher 52)** |
| --- | --- | --- | --- | --- | --- | --- | --- |
| IBDV-PV557493 | 94.26 | 96.17 | 96.17 | 99.57 | 95.32 | 95.54 | 97.23 |
| IBDV-PV557494 | 94.26 | 96.17 | 96.17 | 99.57 | 95.32 | 95.54 | 97.23 |
| IBDV-PV557495 | 94.26 | 96.17 | 96.17 | 99.57 | 95.32 | 95.54 | 97.23 |
| IBDV-PV557496 | 96.17 | 90.87 | 91.5 | 91.29 | 89.59 | 89.8 | 91.93 |
| IBDV-PV557497 | 96.17 | 91.29 | 91.93 | 91.71 | 90.02 | 90.23 | 92.35 |
| IBDV-PV557498 | 96.17 | 90.87 | 91.5 | 91.29 | 89.59 | 89.8 | 91.93 |
| IBDV-PV557499 | 96.39 | 91.08 | 91.71 | 91.5 | 89.8 | 90.02 | 92.14 |
| IBDV-PV557500 | 96.81 | 91.5 | 92.14 | 91.93 | 90.23 | 90.44 | 92.99 |
| IBDV-PV557501 | 96.81 | 91.5 | 92.14 | 91.93 | 90.23 | 90.44 | 92.56 |
| IBDV-PV557502 | 96.39 | 91.5 | 92.14 | 91.93 | 90.65 | 90.87 | 92.56 |
| IBDV-PV557503 | 96.6 | 91.29 | 91.93 | 91.71 | 90.44 | 90.65 | 92.35 |
| IBDV-PV557504 | 97.02 | 91.71 | 92.35 | 92.14 | 90.44 | 90.65 | 92.78 |
| IBDV-PV557505 | 97.02 | 91.71 | 92.35 | 92.14 | 90.44 | 90.65 | 92.78 |
| IBDV-PV557506 | 96.81 | 91.5 | 92.14 | 91.93 | 90.23 | 90.44 | 92.99 |
| IBDV-PV557507 | 96.6 | 91.29 | 91.93 | 91.29 | 90.02 | 90.23 | 92.35 |
| IBDV-PV557508 | 96.6 | 91.71 | 92.35 | 92.14 | 90.87 | 91.08 | 92.78 |
| IBDV-PV557509 | 95.75 | 90.44 | 91.08 | 90.87 | 89.59 | 89.8 | 91.5 |
| IBDV-PV557510 | 96.39 | 91.08 | 91.71 | 91.5 | 90.23 | 90.44 | 92.14 |
| IBDV-PV557511 | 96.17 | 91.29 | 91.93 | 91.71 | 90.44 | 90.65 | 92.35 |
| IBDV-PV557512 | 96.17 | 91.29 | 91.93 | 91.71 | 90.44 | 90.65 | 92.35 |
| IBDV-PV557513 | 96.6 | 91.29 | 91.93 | 91.71 | 90.87 | 91.08 | 92.35 |
| IBDV-PV557514 | 95.75 | 90.87 | 91.5 | 91.29 | 90.23 | 90.44 | 91.93 |
| IBDV-PV557515 | 95.96 | 91.08 | 91.71 | 91.5 | 90.44 | 90.65 | 92.14 |
| IBDV-PV557516 | 95.75 | 90.87 | 91.5 | 91.29 | 90.23 | 90.44 | 91.93 |
| IBDV-PV557517 | 95.54 | 90.23 | 90.87 | 90.65 | 89.38 | 89.59 | 91.29 |
| IBDV-PV557518 | 97.02 | 91.71 | 92.35 | 92.14 | 90.44 | 90.65 | 92.78 |
| IBDV-PV557519 | 96.39 | 91.08 | 91.71 | 91.5 | 89.8 | 90.02 | 92.14 |
| IBDV-PV557520 | 98.3 | 94.26 | 94.9 | 94.26 | 92.78 | 92.99 | 95.32 |
| IBDV-PV557521 | 96.81 | 91.5 | 92.14 | 91.93 | 90.23 | 90.44 | 92.56 |
| IBDV-PV557522 | 96.39 | 91.5 | 92.14 | 91.93 | 90.65 | 90.87 | 92.56 |
| IBDV-PV557523 | 96.81 | 91.5 | 92.14 | 91.93 | 90.23 | 90.44 | 92.56 |
| IBDV-PV557524 | 96.81 | 91.5 | 92.14 | 91.93 | 90.65 | 90.87 | 92.56 |
| IBDV-PV557525 | 96.39 | 91.08 | 91.71 | 91.5 | 90.23 | 90.44 | 92.14 |
| IBDV-PV557526 | 96.39 | 91.5 | 92.14 | 91.93 | 90.65 | 90.87 | 92.99 |
| IBDV-PV557527 | 95.96 | 93.2 | 93.84 | 93.2 | 91.71 | 91.93 | 94.69 |
