# Supplementary material for: Predominance of reassortant infectious bursal disease viruses in Turkish poultry flocks
Source: Poult Sci. 2025 Oct 20;104(12):105974. doi: 10.1016/j.psj.2025.105974 (PMC12747199; doi:10.1016/j.psj.2025.105974)
Supplement: Supplementary file 2 [file mmc2.docx]

Supplementary Table 3: Nucleotide homology analysis of the VP1 gene among the 35 IBDV strains in this study and reference vaccine strains

| **IBDV strains** | **AJ318897.1**  **(UK661)** | **EU162090.1**  **(D78)** | **AJ878657.1 (228E)** | **AF083092.1 (Winterfield 2512)** | **AJ878655.1**  (Bursine 2) | **HG974566.1**  **(Faragher 52)** | **AY918947.1** (Lukert) |
| --- | --- | --- | --- | --- | --- | --- | --- |
| IBDV-PV557458 | 88.36 | 97.22 | 81.42 | 97.04 | 95.31 | 95.31 | 80.38 |
| IBDV-PV557459 | 88.19 | 97.39 | 81.59 | 97.22 | 95.13 | 95.13 | 80.2 |
| IBDV-PV557460 | 88.36 | 97.22 | 81.42 | 97.04 | 94.96 | 94.96 | 80.03 |
| IBDV-PV557461 | 88.02 | 96.52 | 80.9 | 96.35 | 94.27 | 94.27 | 79.68 |
| IBDV-PV557462 | 88.02 | 97.74 | 81.59 | 97.56 | 95.65 | 96.18 | 81.07 |
| IBDV-PV557463 | 88.19 | 96.7 | 81.07 | 96.52 | 94.44 | 94.44 | 79.86 |
| IBDV-PV557464 | 88.19 | 96.7 | 81.07 | 96.52 | 94.44 | 94.44 | 79.86 |
| IBDV-PV557465 | 88.19 | 96.87 | 81.42 | 96.7 | 94.96 | 94.96 | 80.03 |
| IBDV-PV557466 | 87.67 | 96.35 | 81.25 | 96.18 | 94.44 | 94.44 | 79.86 |
| IBDV-PV557467 | 88.36 | 97.22 | 81.42 | 97.04 | 94.96 | 94.96 | 80.03 |
| IBDV-PV557468 | 87.67 | 97.04 | 81.25 | 96.87 | 94.79 | 94.79 | 79.86 |
| IBDV-PV557469 | 83.68 | 92.01 | 77.6 | 91.84 | 90.27 | 90.1 | 76.56 |
| IBDV-PV557470 | 87.67 | 97.04 | 81.25 | 96.87 | 94.79 | 94.79 | 79.86 |
| IBDV-PV557471 | 88.19 | 97.22 | 81.42 | 97.04 | 95.31 | 95.31 | 80.38 |
| IBDV-PV557472 | 84.72 | 92.88 | 78.12 | 92.7 | 90.97 | 90.79 | 76.9 |
| IBDV-PV557473 | 87.67 | 97.04 | 81.25 | 96.87 | 94.79 | 94.79 | 79.86 |
| IBDV-PV557474 | 87.67 | 97.04 | 81.25 | 96.87 | 94.79 | 94.79 | 79.86 |
| IBDV-PV557475 | 87.84 | 97.04 | 81.25 | 96.87 | 94.79 | 94.79 | 79.86 |
| IBDV-PV557476 | 88.19 | 97.22 | 81.25 | 97.04 | 94.96 | 94.96 | 79.86 |
| IBDV-PV557477 | 88.19 | 97.22 | 81.42 | 97.04 | 94.96 | 94.96 | 80.03 |
| IBDV-PV557478 | 88.02 | 97.04 | 81.42 | 96.87 | 94.79 | 94.79 | 80.03 |
| IBDV-PV557479 | 88.19 | 97.22 | 81.42 | 97.04 | 95.31 | 95.31 | 80.38 |
| IBDV-PV557480 | 88.71 | 98.09 | 82.46 | 97.91 | 98.09 | 98.61 | 83.15 |
| IBDV-PV557481 | 88.71 | 98.09 | 81.77 | 97.91 | 97.39 | 97.91 | 82.46 |
| IBDV-PV557482 | 88.02 | 97.39 | 81.94 | 97.22 | 97.39 | 97.91 | 82.63 |
| IBDV-PV557483 | 88.19 | 97.39 | 81.59 | 97.22 | 95.13 | 95.13 | 80.2 |
| IBDV-PV557484 | 97.39 | 88.54 | 73.43 | 88.36 | 88.02 | 88.36 | 73.61 |
| IBDV-PV557485 | 97.74 | 88.36 | 73.43 | 88.19 | 87.84 | 88.19 | 73.61 |
| IBDV-PV557486 | 87.84 | 97.22 | 81.42 | 97.04 | 94.96 | 94.96 | 80.03 |
| IBDV-PV557487 | 88.02 | 96.87 | 81.07 | 96.7 | 94.61 | 94.61 | 80.03 |
| IBDV-PV557488 | 88.19 | 97.04 | 81.25 | 96.87 | 95.13 | 95.13 | 80.2 |
| IBDV-PV557489 | 88.19 | 96.87 | 81.07 | 96.7 | 94.61 | 94.61 | 79.68 |
| IBDV-PV557490 | 88.19 | 97.56 | 81.59 | 97.39 | 95.31 | 95.31 | 80.2 |
| IBDV-PV557491 | 85.24 | 93.57 | 78.47 | 93.4 | 91.66 | 91.66 | 77.43 |
| IBDV-PV557492 | 85.24 | 93.75 | 78.64 | 93.57 | 91.84 | 91.84 | 77.6 |
