# Supplementary material for: Predominance of reassortant infectious bursal disease viruses in Turkish poultry flocks
Source: Poult Sci. 2025 Oct 20;104(12):105974. doi: 10.1016/j.psj.2025.105974 (PMC12747199; doi:10.1016/j.psj.2025.105974)
Supplement: Supplementary file 3 [file mmc3.docx]

Supplementary Table 4: Amino acid sequence analysis of the VP2 protein across the 35 IBDV strains and reference vaccine strains

| **IBDV strains** | **NC_004178.1**  **(UK661)** | **EU162087.1**  **(D78)** | **AJ586966.1**  **(228E)** | **MH329181.1**  **(Winterfield 2512)** | **AY918948.1**  (Lukert) | **AF498631.1**  (Bursine 2) | **HG974565.1**  **(Faragher 52)** |
| --- | --- | --- | --- | --- | --- | --- | --- |
| IBDV-PV557493 | 96.17 | 94.9 | 95.54 | 100 | 92.35 | 92.99 | 97.45 |
| IBDV-PV557494 | 96.17 | 94.9 | 95.54 | 100 | 92.35 | 92.99 | 97.45 |
| IBDV-PV557495 | 96.17 | 94.9 | 95.54 | 100 | 92.35 | 92.99 | 97.45 |
| IBDV-PV557496 | 96.81 | 90.44 | 92.99 | 92.99 | 89.17 | 89.8 | 94.26 |
| IBDV-PV557497 | 97.45 | 91.08 | 93.63 | 93.63 | 89.8 | 90.44 | 94.9 |
| IBDV-PV557498 | 96.81 | 90.44 | 92.99 | 92.99 | 89.17 | 89.8 | 94.26 |
| IBDV-PV557499 | 96.81 | 90.44 | 92.99 | 92.99 | 89.17 | 89.8 | 94.26 |
| IBDV-PV557500 | 97.45 | 91.08 | 93.63 | 93.63 | 89.8 | 90.44 | 94.9 |
| IBDV-PV557501 | 97.45 | 91.08 | 93.63 | 93.63 | 89.8 | 90.44 | 94.9 |
| IBDV-PV557502 | 96.81 | 91.71 | 94.26 | 94.26 | 90.44 | 91.08 | 95.54 |
| IBDV-PV557503 | 96.81 | 90.44 | 92.99 | 92.99 | 89.17 | 89.8 | 94.26 |
| IBDV-PV557504 | 97.45 | 91.08 | 93.63 | 93.63 | 89.8 | 90.44 | 94.9 |
| IBDV-PV557505 | 97.45 | 91.08 | 93.63 | 93.63 | 89.8 | 90.44 | 94.9 |
| IBDV-PV557506 | 97.45 | 91.08 | 93.63 | 93.63 | 89.8 | 90.44 | 94.9 |
| IBDV-PV557507 | 96.17 | 90.44 | 92.99 | 92.99 | 89.17 | 89.8 | 94.26 |
| IBDV-PV557508 | 96.81 | 91.71 | 94.26 | 94.26 | 90.44 | 91.08 | 95.54 |
| IBDV-PV557509 | 94.9 | 88.53 | 91.08 | 91.08 | 87.26 | 87.89 | 92.35 |
| IBDV-PV557510 | 96.81 | 90.44 | 92.99 | 92.99 | 89.17 | 89.8 | 94.26 |
| IBDV-PV557511 | 97.45 | 91.08 | 93.63 | 93.63 | 89.8 | 90.44 | 94.9 |
| IBDV-PV557512 | 97.45 | 91.08 | 93.63 | 93.63 | 89.8 | 90.44 | 94.9 |
| IBDV-PV557513 | 97.45 | 91.08 | 93.63 | 93.63 | 89.8 | 90.44 | 94.9 |
| IBDV-PV557514 | 97.45 | 91.08 | 93.63 | 93.63 | 89.8 | 90.44 | 94.9 |
| IBDV-PV557515 | 97.45 | 91.08 | 93.63 | 93.63 | 89.8 | 90.44 | 94.9 |
| IBDV-PV557516 | 97.45 | 91.08 | 93.63 | 93.63 | 89.8 | 90.44 | 94.9 |
| IBDV-PV557517 | 94.9 | 88.53 | 91.08 | 91.08 | 87.26 | 87.89 | 92.35 |
| IBDV-PV557518 | 97.45 | 91.08 | 93.63 | 93.63 | 89.8 | 90.44 | 94.9 |
| IBDV-PV557519 | 96.17 | 89.8 | 92.35 | 92.35 | 88.53 | 89.17 | 93.63 |
| IBDV-PV557520 | 98.72 | 92.35 | 94.9 | 94.9 | 91.08 | 91.71 | 96.17 |
| IBDV-PV557521 | 97.45 | 91.08 | 93.63 | 93.63 | 89.8 | 90.44 | 94.9 |
| IBDV-PV557522 | 97.45 | 91.08 | 93.63 | 93.63 | 89.8 | 90.44 | 94.9 |
| IBDV-PV557523 | 97.45 | 91.08 | 93.63 | 93.63 | 89.8 | 90.44 | 94.9 |
| IBDV-PV557524 | 97.45 | 91.08 | 93.63 | 93.63 | 89.8 | 90.44 | 94.9 |
| IBDV-PV557525 | 97.45 | 91.08 | 93.63 | 93.63 | 89.8 | 90.44 | 94.9 |
| IBDV-PV557526 | 96.81 | 91.71 | 94.26 | 94.26 | 90.44 | 91.08 | 95.54 |
| IBDV-PV557527 | 94.9 | 89.8 | 92.35 | 92.35 | 88.53 | 89.17 | 93.63 |
