# Supplementary material for: Predominance of reassortant infectious bursal disease viruses in Turkish poultry flocks
Source: Poult Sci. 2025 Oct 20;104(12):105974. doi: 10.1016/j.psj.2025.105974 (PMC12747199; doi:10.1016/j.psj.2025.105974)
Supplement: Supplementary file 4 [file mmc4.docx]

Supplementary Table 5: Amino acid sequence analysis of the VP1 protein across the 35 IBDV strains and reference vaccine strains

| **IBDV strains** | **AJ318897.1**  **(UK661)** | **EU162090.1**  **(D78)** | **AJ878657.1 (228E)** | **AF083092.1 (Winterfield 2512)** | **AJ878655.1**  (Bursine 2) | **HG974566.1**  **(Faragher 52)** | **AY918947.1** (Lukert) |
| --- | --- | --- | --- | --- | --- | --- | --- |
| IBDV-PV557458 | 96.87 | 99.47 | 98.76 | 99.47 | 97.91 | 98.95 | 98.76 |
| IBDV-PV557459 | 96.87 | 99.47 | 98.76 | 99.47 | 97.91 | 98.95 | 98.76 |
| IBDV-PV557460 | 96.87 | 99.47 | 98.76 | 99.47 | 97.91 | 98.95 | 98.76 |
| IBDV-PV557461 | 96.35 | 98.95 | 98.14 | 98.95 | 97.39 | 98.43 | 98.14 |
| IBDV-PV557462 | 96.87 | 98.95 | 98.14 | 98.95 | 97.39 | 98.43 | 98.14 |
| IBDV-PV557463 | 96.87 | 99.47 | 98.76 | 99.47 | 97.91 | 98.95 | 98.76 |
| IBDV-PV557464 | 96.87 | 99.47 | 98.76 | 99.47 | 97.91 | 98.95 | 98.76 |
| IBDV-PV557465 | 96.35 | 98.95 | 98.14 | 98.95 | 97.39 | 98.43 | 98.14 |
| IBDV-PV557466 | 95.31 | 97.91 | 98.14 | 97.91 | 96.35 | 97.39 | 98.14 |
| IBDV-PV557467 | 96.87 | 99.47 | 98.76 | 99.47 | 97.91 | 98.95 | 98.76 |
| IBDV-PV557468 | 96.35 | 98.95 | 98.14 | 98.95 | 97.39 | 98.43 | 98.14 |
| IBDV-PV557469 | 86.45 | 89.06 | 90.12 | 89.06 | 88.54 | 88.54 | 90.12 |
| IBDV-PV557470 | 96.87 | 99.47 | 98.76 | 99.47 | 97.91 | 98.95 | 98.76 |
| IBDV-PV557471 | 96.87 | 99.47 | 98.76 | 99.47 | 97.91 | 98.95 | 98.76 |
| IBDV-PV557472 | 88.02 | 90.62 | 90.74 | 90.62 | 90.1 | 90.1 | 91.35 |
| IBDV-PV557473 | 96.35 | 98.95 | 98.14 | 98.95 | 97.39 | 98.43 | 98.14 |
| IBDV-PV557474 | 96.35 | 98.95 | 98.14 | 98.95 | 97.39 | 98.43 | 98.14 |
| IBDV-PV557475 | 96.87 | 99.47 | 98.76 | 99.47 | 97.91 | 98.95 | 98.76 |
| IBDV-PV557476 | 96.35 | 98.95 | 98.14 | 98.95 | 97.39 | 98.43 | 98.14 |
| IBDV-PV557477 | 96.87 | 99.47 | 98.76 | 99.47 | 97.91 | 98.95 | 98.76 |
| IBDV-PV557478 | 95.83 | 98.43 | 98.14 | 98.43 | 96.87 | 97.91 | 98.14 |
| IBDV-PV557479 | 96.87 | 99.47 | 98.76 | 99.47 | 97.91 | 98.95 | 98.76 |
| IBDV-PV557480 | 97.39 | 99.47 | 98.76 | 99.47 | 97.91 | 98.95 | 98.76 |
| IBDV-PV557481 | 97.39 | 99.47 | 98.76 | 99.47 | 97.91 | 98.95 | 98.76 |
| IBDV-PV557482 | 95.83 | 97.91 | 97.53 | 97.91 | 96.35 | 97.39 | 97.53 |
| IBDV-PV557483 | 96.87 | 99.47 | 98.76 | 99.47 | 97.91 | 98.95 | 98.76 |
| IBDV-PV557484 | 97.91 | 97.91 | 97.53 | 97.91 | 96.35 | 97.39 | 97.53 |
| IBDV-PV557485 | 98.43 | 97.39 | 96.91 | 97.39 | 95.83 | 96.87 | 96.91 |
| IBDV-PV557486 | 96.87 | 99.47 | 98.76 | 99.47 | 97.91 | 98.95 | 98.76 |
| IBDV-PV557487 | 96.35 | 98.95 | 98.14 | 98.95 | 97.39 | 98.43 | 98.14 |
| IBDV-PV557488 | 96.35 | 98.95 | 98.14 | 98.95 | 97.39 | 98.43 | 98.14 |
| IBDV-PV557489 | 96.35 | 98.43 | 97.53 | 98.43 | 96.87 | 97.91 | 97.53 |
| IBDV-PV557490 | 96.87 | 99.47 | 98.76 | 99.47 | 97.91 | 98.95 | 98.76 |
| IBDV-PV557491 | 88.02 | 90.62 | 91.97 | 90.62 | 89.06 | 90.1 | 91.97 |
| IBDV-PV557492 | 88.54 | 91.14 | 92.59 | 91.14 | 89.58 | 90.62 | 92.59 |
